# Supplementary material for: A monolithically integrated polarization entangled photon pair source on a silicon chip
Source: Sci Rep. 2012 Nov 12;2:817. doi: 10.1038/srep00817 (PMC3495342; doi:10.1038/srep00817)
Supplement: Supplementary Information [file srep00817-s1.pdf]

Supplementary information:

"A monolithically-integrated polarization entangled photon pair source on a silicon chip"

Nobuyuki Matsuda[1,3], Hanna Le Jeannic[1], Hiroshi Fukuda[2,3], Tai Tsuchizawa[2,3], William John Munro[1], Kaoru Shimizu[1], Koji Yamada[2,3], Yasuhiro Tokura[1], Hiroki Takesue[1]

[1]NTT Basic Research Laboratories, NTT Corporation, Atsugi, Kanagawa 243-0198, Japan

[2]NTT Microsystem Integration Laboratories, NTT Corporation, Atsugi, Kanagawa 243-0198, Japan

[3]Nanophotonics Center, NTT Corporation, Atsugi, Kanagawa 243-0198, Japan

- Polarization dependence of FWM efficiency in a silicon-wire waveguide

In Fig. S1, we see an almost straight line consisting of created idler peaks, which appear almost symmetric with the signal line with respect to the pump wavelength. This is explained by the energy conservation of the FWM. The clear idler peak was observed only for the TE-polarized FWM, due to a strong field confinement in the core (Fig. S2) and the near-zero-dispersion property of the TE mode in the telecom band (Fig. S3). The data in Fig. 2a was extracted from the data in Fig. S1.

- Imperfection in obtained fully entangled fraction $F(\rho_{ent})$

First, to verify the reproducibility of the experiment we measured ρ_{ent} four times, and on each occasion we reset the input pump polarization manually using the input half-wave plate. The average value and the standard error were $F(\rho_{ent}) = 0.91 \pm 0.02$, which means that the angular settings of the wave plates were sufficiently reproducible. Second, the value of $F(\rho_{ent})$ without accidental coincidence counts was obtained as 0.92 ± 0.02, which indicates that statistical background coincidence events contribute little to the degraded fidelity.

The density matrix ρ_{ref} in Fig. 4a exhibits $|TE,TM>_{s,i}$ and $|TM,TE>_{s,i}$ components, implying that the generated $|TE,TE>_{s,i}$ pairs in the reference silicon-wire waveguide suffered slight polarization rotation. We investigated this for the signal and idler wavelength modes using a classical amplified-spontaneous-emission source. The light

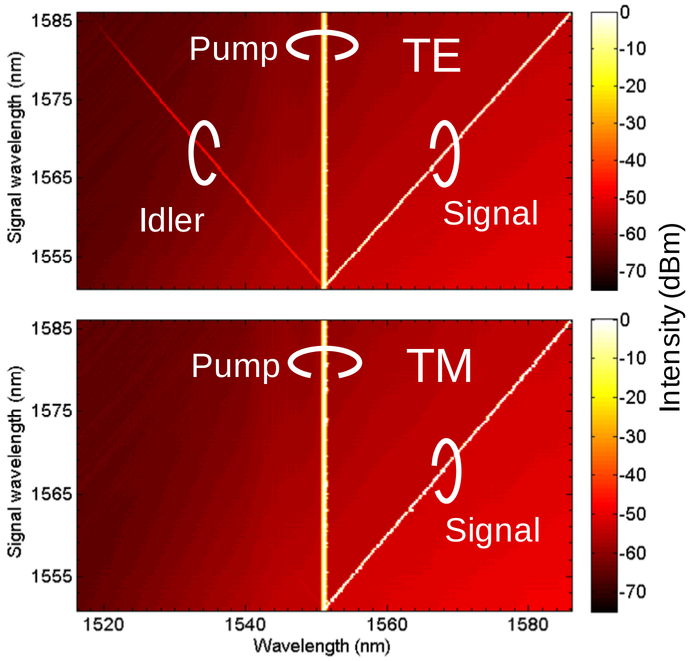

Figure S1 Polarization dependence of the FWM efficiency in a silicon-wire waveguide investigated with a stimulated FWM experiment. Each density plot shows the observed FWM spectrum as a function of the signal wavelength (vertical axis).

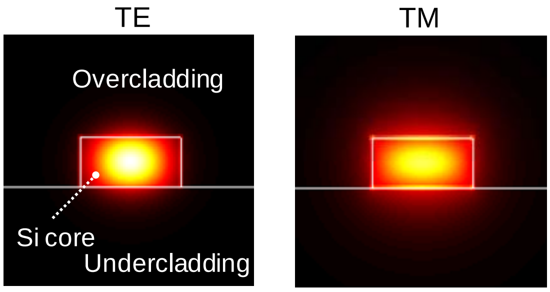

Figure S2 Field distribution of the fundamental modes in the silicon-wire waveguide, calculated with a mode solver.

source passed through the WDM filter before being coupled to the reference silicon-wire waveguide so that its spectrum was identical to that of the filtered photon pairs. Then, the polarization rotation property of the device was investigated (Fig. S4) in the same way as for the pump wavelength. As can be seen, there is a horizontal offset in the fringes for the signal and idler modes, even without the silicon polarization rotator. The unexpected polarization rotation might be due to a fabrication error, which is

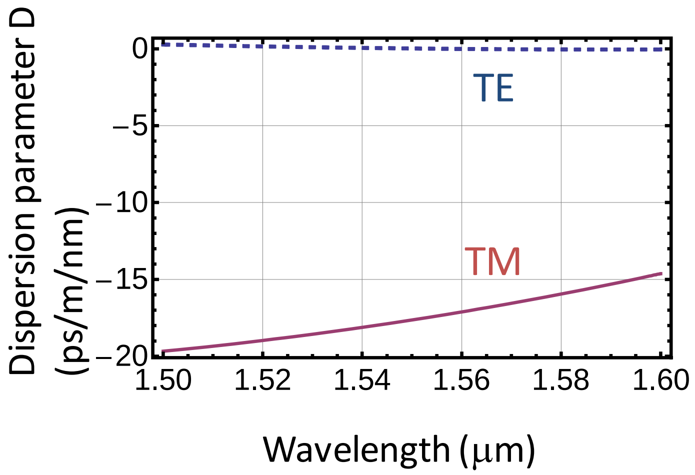

Figure S3 Calculated polarization-dependent dispersion property in the silicon-wire waveguide.

presumably a slight horizontal offset between the axis of the inverse-taper silicon wire and the second core at the SSCs. The amplified-spontaneous-emission source centred at the signal and the idler wavelengths had polarization rotations of − 11.0 ± 0.2° and − 9.9 ± 0.2°, respectively. If these values are contributed by the SSCs at the input and the output equally, the created $|TE,TE>_{s,i}$ pairs in the reference SWW can be expected to suffer a polarization rotation of approximately − 6° at the output SSC. We plot the estimated density matrix for this case in Fig. S5, which well describes the feature of the experimentally obtained ρ_{ref} (Fig. 4a). In addition we can also see the wavelength-dependent polarization rotation at the silicon polarization rotator.

These insufficient polarization rotations cause the propagating optical field to have a slightly rotated linearly polarized state from either the TE or TM mode that suffers from the strong birefringence of the following silicon-wire waveguides. As a result, the propagating field exhibits a rapid oscillation of the polarization state in the spectral region [32]. Such polarization oscillation is averaged out in the WDM filter window for the signal and idler modes, causing the degree of polarization to degrade. Therefore, we obtained the degraded fringe visibility seen in Fig. S4 and also an imperfect $F(\rho_{ent})$ value. We obtained a fringe visibility of 0.92. $F(\rho_{ent})$ could degrade to the same degree.

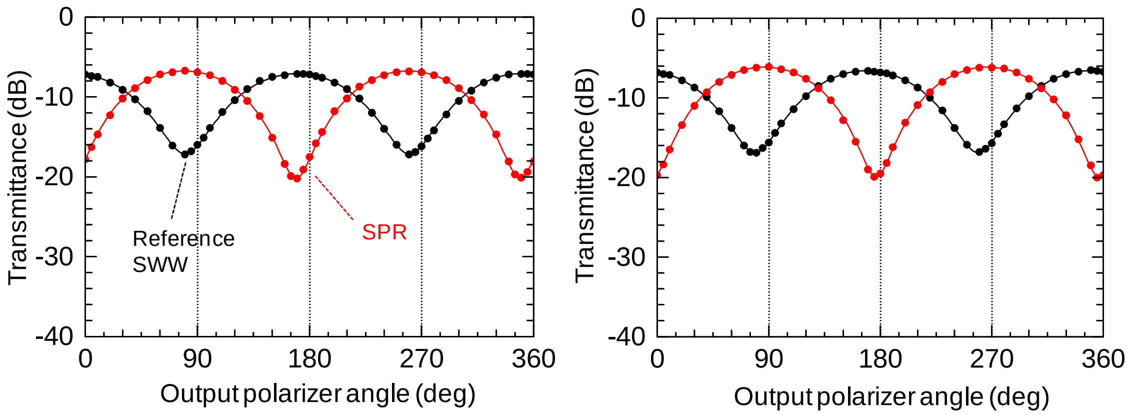

Figure S4 Measured transmittance as a function of the output polarizer angle at the signal (left) and idler (right) wavelengths.

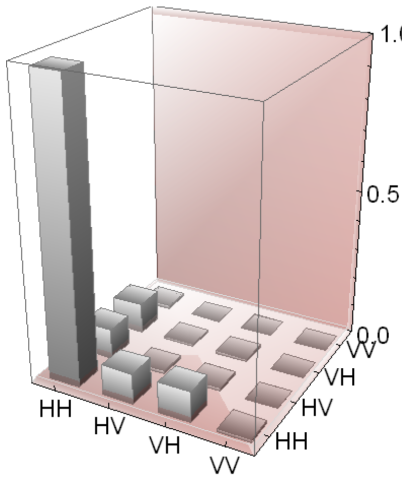

Figure S5 Calculated absolute values of the elements of ρ_{ref} in the presence of the finite polarization rotation at the output SSC. H and V represent the TE and TM modes, respectively.
